# Supplementary material for: Enteric Coronavirus Infection and Treatment Modeled With an Immunocompetent Human Intestine-On-A-Chip
Source: Front Pharmacol. 2021 Oct 25;12:718484. doi: 10.3389/fphar.2021.718484 (PMC8573067; doi:10.3389/fphar.2021.718484)
Supplement: Supplementary file 1 [file DataSheet1.PDF]

SYBR green primers

Primer	Forward	Reverse
NL63 subgenomic	GATAGAGAATTTTCTTATTTAGACTTTGTG	CATGTAAAATGAAGGAGGAGGAA
GAPDH	TGCACCACCAACTGCTTAGC	GGCATGGACTGTGGTCATGAG

Taqman primers

Primer	Catalog Number (Thermo Fisher)
OC43	Vi06439646_s1
ACE2	Hs01085333_m1
LGR5	Hs00969422_m1
ACTB	Hs01060665_g1
GAPDH	Hs02786624_g1
TMPRSS2	Hs01122322_m1
TMPRSS4	Hs00854071_mH
FURIN	Hs00965485_g1

Supplementary Figure 1

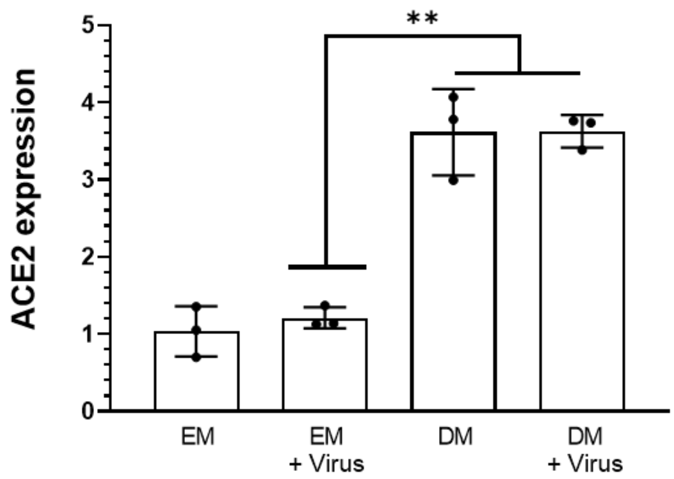

Supplementary Figure 2

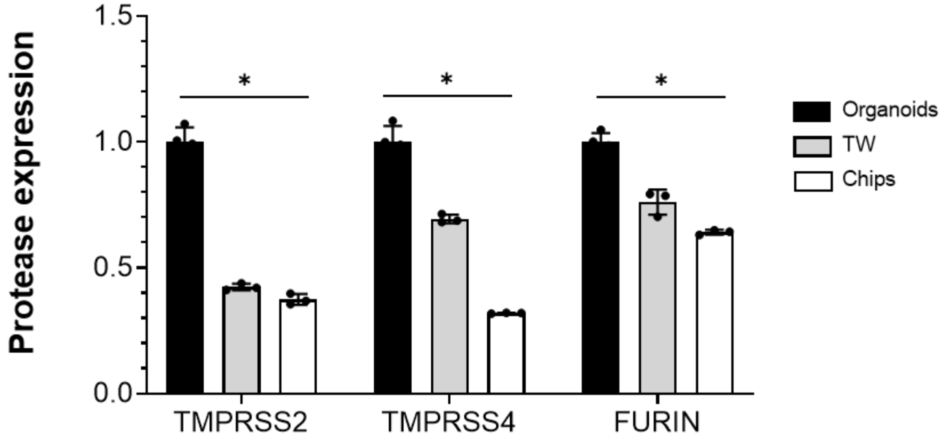

Supplementary Figure 3

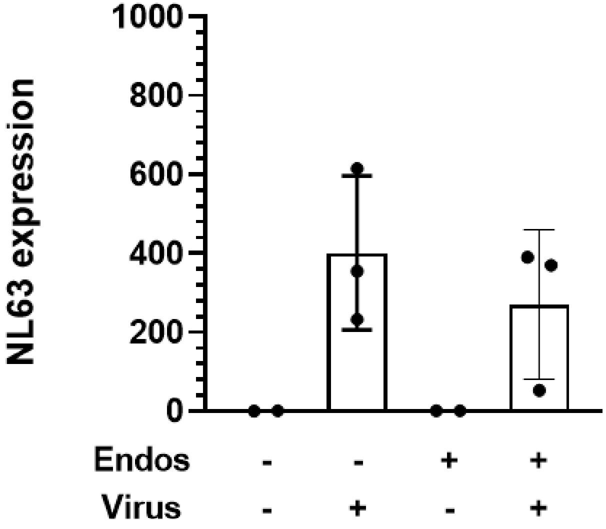

Supplementary Figure 4

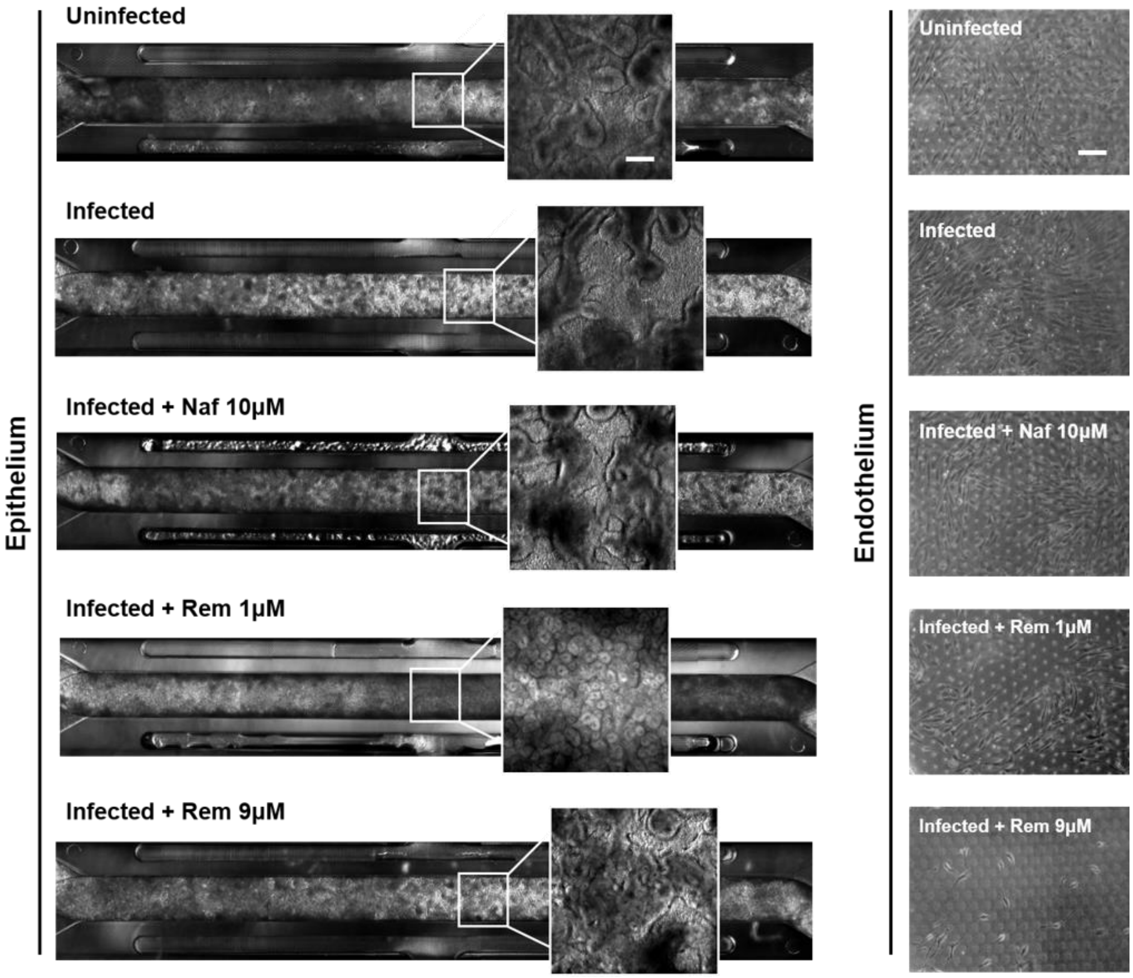

Supplementary Figure 5

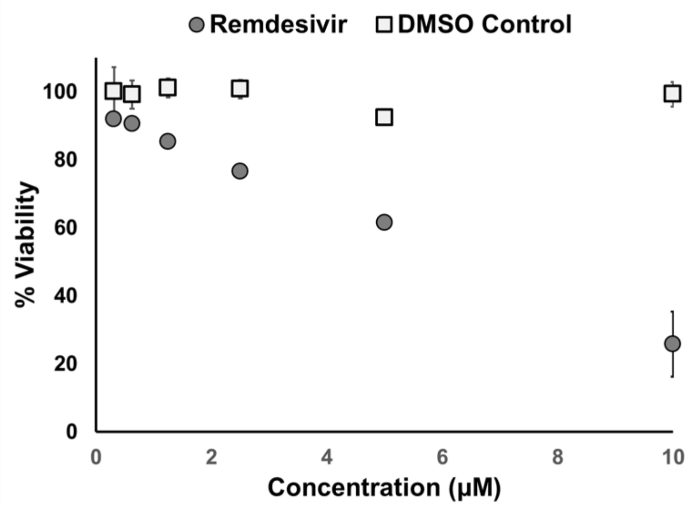

Supplementary Figure 6

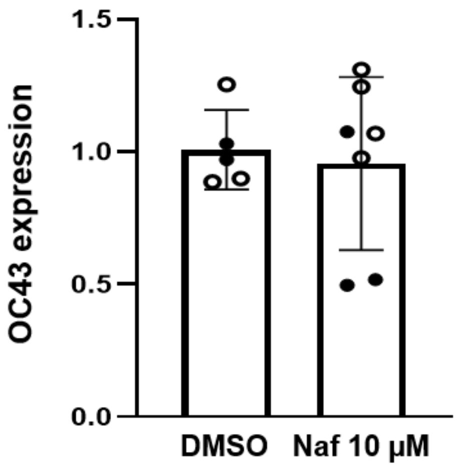

Supplementary Figure 7
